# Triple Negative Breast Cancer Profile, from Gene to microRNA, in Relation to Ethnicity

**DOI:** 10.3390/cancers11030363

**Published:** 2019-03-13

**Authors:** Ishita Gupta, Rasha M. Sareyeldin, Israa Al-Hashimi, Hamda A. Al-Thawadi, Halema Al Farsi, Semir Vranic, Ala-Eddin Al Moustafa

**Affiliations:** 1 College of Medicine, Qatar University, Doha P.O. Box: 2713, Qatar; ishugupta28@gmail.com (I.G.); rs1600253@student.qu.edu.qa (R.M.S.); ia1507081@student.qu.edu.qa (I.A.-H.); halthawadi@qu.edu.qa (H.A.A.-T.); halfarsi@qu.edu.qa (H.A.F.); semir.vranic@gmail.com (S.V.); 2 Biomedical Research Centre, Qatar University, Doha P.O. Box: 2713, Qatar

**Keywords:** breast cancer, triple negative breast cancer, biomarkers, microarray, gene expression profiling, miRNA

## Abstract

Breast cancer is the most frequent cause of cancer-related deaths among women worldwide. It is classified into four major molecular subtypes. Triple-negative breast cancers (TNBCs), a subgroup of breast cancer, are defined by the absence of estrogen and progesterone receptors and the lack of HER-2 expression; this subgroup accounts for ~15% of all breast cancers and exhibits the most aggressive metastatic behavior. Currently, very limited targeted therapies exist for the treatment of patients with TNBCs. On the other hand, it is important to highlight that knowledge of the molecular biology of breast cancer has recently changed the decision-making process regarding the course of cancer therapies. Thus, a number of new techniques, such as gene profiling and sequencing, proteomics, and microRNA analysis have been used to explore human breast carcinogenesis and metastasis including TNBC, which consequently could lead to new therapies. Nevertheless, based on evidence thus far, genomics profiles (gene and miRNA) can differ from one geographic location to another as well as in different ethnic groups. This review provides a comprehensive and updated information on the genomics profile alterations associated with TNBC pathogenesis associated with different ethnic backgrounds.

## 1. Introduction

Breast cancer is the most frequently diagnosed cancer in women worldwide [1]. In 2012, breast cancer accounted for 25% of the prevalent cancer cases worldwide [2]. In developing countries, it is the most common cause of death (14.3%), whereas in developed countries it is the second leading cause of cancer mortality (15.4%) [1].

Various environmental factors contribute to a woman′s risk of developing breast cancer. Increasing age, menarche, high hormonal levels, null-parity, tobacco use, and obesity [3,4,5,6,7,8,9] are risk factors and account for 47% of the breast cancer (BC) cases [10]. Approximately 5–10% of the cases are attributed to genetic factors that include *BRCA* (*BRCA1* and *BRCA2*) mutations [11,12,13]. *BRCA1*/2 are autosomal dominant and tumor suppressor genes present on chromosomes 17 and 13, respectively, and are mutated in approximately 30–40% of familial BC cases [14].

On the other hand, oncogenes and tumor suppressor genes are involved in the tumorigenesis of sporadic BC [15]. While most of cancer-related deaths are a result of complications from its metastatic form [16,17]; however, the mechanisms underlying malignant progression in BC are yet to be elucidated. Research has identified numerous genetic changes in malignant tumors, although the frequency of different gene alterations is quite low [18]. Recently, “significantly mutated genes” (SMGs) were identified in the onset of malignant transformation [19] and few of them encode for proteins interacting with *BRCA1/2*, while others act through different pathways including *TP53*, *PTEN*, *CHEK2*, *ATM* and *PALB2* [20]. Mutations in these genes are suspected to elevate the risk of BC development.

Various prognostic and predictive factors are studied in BC, including estrogen/progesterone receptors (ER/PR) status and *HER-2/neu* gene amplification [21,22]. Steroid receptor status, HER-2/neu status, nodal status, tumor size, and grade have been used for several years [23], however, none of these factors are reliable predictors of disease outcome.

Gene expression profiling in BC started in the mid-1990s, this technique allowed classification of BC into subtypes via hierarchical clustering of several gene expression profiles of human breast tumors [24,25,26]. BC was first classified into its intrinsic molecular subtypes luminal, Her2, basal-like and normal breast using cDNA microarrays by Perou and colleagues (2000) [27]. Following this study, another study differentiated molecular subtypes linked with different prognosis and further subdivided the luminal group into luminal A and luminal B [28]. Analysis between the subtypes showed the basal-like and the Her2+ subtypes have the shortest overall survival times and relapse-free survival in comparison with the estrogen-receptor positive groups [29]. The study showed that the basal-like subtype potentially represented a different clinical entity linked with shorter survival and a high frequency of *TP53* mutations. Genome-wide expression arrays of tumors demonstrated the tumor biology; range in patterns reflected the biological diversity [29]. Based on these subtypes, an Expert Consensus established four clinic-pathological definitions, recommending therapeutic strategies for each group [30]. Further research revealed additional subtypes such as a claudin-low BC, a subtype of basal-like BC [31]. However, a larger cohort of breast tumors needs to be assessed along with comprehensive clinical information to identify clinical phenotypes including resistance and sensitivity to specific therapies, invasiveness, or metastatic potential [29].

In this review, we will focus on the role of microarray molecular profiling (genes and microRNAs) as a prognostic, diagnostic as well as a therapeutic tool for the most aggressive BC phenotype in different ethnic groups, which is triple negative BC.

## 2. Triple Negative Breast Cancer (TNBC)

Triple negative breast cancer (TNBC) is a subgroup of BC, representing 12–17% of all BCs [32]. TNBCs have a comparatively lower expression of the three receptors: ER, PR and HER-2/neu in comparison with normal tissue as well as other types of BC. It affects more frequently young patients, and is represented by advanced stage, higher proliferative index (measured by mitotic account or Ki-67 proliferative index), higher histologic grade, and significantly higher metastatic rates [33,34,35,36].

TNBCs have a higher prevalence in a distinct group or population [13]; for example, in African-American women the prevalence of TNBCs is very high [37]. TNBC was found to be prevalent in young women of African descent [38]. Environmental as well as genetic factors are known to impact the age of onset and subtype frequency in different populations [38]. In TNBCs, metastatic rates are high to visceral organs [39,40]; in addition, cerebral metastasis is more common [17,41,42,43]. De-novo metastasis plays a key role in cancer mortality with racial/ethnic disparities in the site, frequency, and associated survival [44]. Racial/ethnic differences in BC can partially be due to variations in the biological aggressiveness of TNBC in African women as compared with other racial/ethnic groups [45]. Recent studies in BC patients showed that non-Hispanic blacks largely had metastasis to the bone, brain, or liver, while Hispanics were less likely to have metastasis to the liver in comparison to the non-Hispanic Whites [44].

Sub-classification of TNBCs have been attempted based on several biomarkers including epidermal growth factor receptor (EGFR), vascular endothelial growth factor (VEGF), c-kit and basal cytokeratins (e.g., CK5/6, CK14, CK17), *TP53*, *TOP-2A*, Ki67, Cox-2 and heat shock protein 90 [36]. Nevertheless, all TNBCs have a poor clinical prognosis and special pathological characteristics compared to other subtypes of BC. The overall 5-year survival rate for TNBC is 50–60% [37,46,47], with a lower likelihood of developing recurrent tumor over the following 5-years in these patients [37,48]. TNBCs are associated with a higher rate of local recurrence during the first three years after treatment and a high five-year mortality rate compared with other subtypes of BC [49].

**Figure 1:**
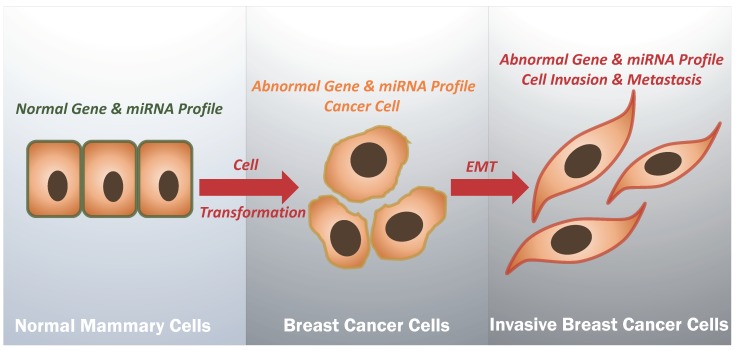
Schematic outline showing normal and abnormal genes and miRNA profiles of normal mammary and breast cancer. It is evident that there are variations in gene expressions and miRNA profiles from normal to non-invasive and invasive cancer, in which epithelial-mesenchymal transition (EMT) is the main hallmark. Thus, combined gene and miRNA profiles can be used as novel Biomarkers and therapy targets for each step of cancer progression. However, it is important to highlight that Gene and miRNA profiles can differ from one geographic location to another as well as between different ethnic groups.

Systemic treatment for breast cancer includes the use of cytotoxic, hormonal, and immunotherapeutic agents. To date, cytotoxic chemotherapy is the only approved treatment option for TNBC [36,50,51]. Systemic agents are effective at the beginning of therapy in the majority (90%) of primary and approximately half of metastatic breast cancer cases [52]. However, after a period of time, tumor progression occurs; resistance to therapy is common leading to treatment failure and death in more than 90% of patients with advanced/metastatic disease [52]. Metastasis is a multifarious process in which a primary solid tumor plagues the adjacent tissue and then spreads to the neighboring as well as distant parts of the body [53]. During tumor progression, the cells undergo epithelial-to-mesenchymal transition (EMT), thus enhancing cell invasion and commencing the process of metastasis, one of the hallmarks of cancer [54] (Figure 1).

Generally, breast cancer cells metastasize to the bone, liver, lung and brain [16]. However, there is no efficient targeted therapy available presently for the treatment of patients with TNBCs, especially in its metastatic form [55].

Knowledge of molecular biology in breast cancer has recently introduced new-targeted therapies using cDNA microarray, proteomics, next-generation sequencing (NGS) and miRNA technologies. Among the novel treatment agents for breast cancer are poly (ADP-ribose) polymerase (PARP) inhibitors, angiogenesis inhibitors, EGFR-targeted agents, and src kinase inhibitors [56]. Other favorable molecular targets include the androgen receptor (AR), insulin-like growth factor receptor (IGFR), protein kinase B (AKT), mTOR [57], PI3K [58] and cyclin-dependent kinases [59].

The following sections will present a comprehensive review about gene expression profiling performed on TNBC to identify potential biomarkers related to cancer progression and metastasis in TNBC patients.

## 3. Gene Expression Profiling of TNBC

Microarray technologies have transformed research, allowing high-throughput whole-genome expression profiling and helped cancer scientists including oncologist to provide insight in a single assay about several diseases as well as create a molecular profile of tumor progression [24,25].

Although on a morphological level TNBC and basal-like breast cancer (BLBC) are comparatively similar in relation to large tumor size, high histologic grade, and substantial metastatic potential [60,61], gene expression profiling classified around 70% of TNBC samples as basal-like [62].

Molecular heterogeneity of TNBC has been recently well characterized at gene expression profiling level. An earlier investigation identified six molecular subtypes of TNBC including basal-like 1, basal-like 2, immunomodulatory, mesenchymal-like, mesenchymal stem-like, and luminal androgen receptor (LAR) subtype [63]. Nevertheless, molecular subtyping of TNBC by gene expression profiling revealed three subtypes, namely luminal androgen receptor, basal-like with low immune response and high M2-like macrophages and, basal-enriched with high immune response and low M2-like macrophages) [64]; which could provide insight for treatment of TNBC.

Both basal-like subtypes (basal-like 1 and basal-like 2) are affected by molecular alterations in cell-cycle, DNA machinery, cell proliferation, glycolysis and gluconeogenesis. These TNBC subtypes were found to be sensitive to cisplatin and PARP inhibitors. However, while, the basal-like 1 subtype displays elevated levels of Ki-67 as well as genes involved in cell division and DNA-damage (*ATR*, *BRCA*, *Myc*, *NRAS*), basal-like 2 subtype is characterized by high levels of *EGFR*, *MET*, *EPHA2* and *TP53* genes [57].

On the other hand, the immunomodulatory subtype was shown to overexpress genes involved in regulating immune cell signaling such as *JAK1/2*, *STAT1/4*, *IRF1/7/8* and *TNF*. Recently, research showed stimulation of the immune signaling pathways including TNF enhanced PD-L1 expression [65]. PD-L1 overexpression is common in basal breast cancers and is linked with high T-cell cytotoxic immune response, better survival and response to chemotherapy [65,66]. The gene expression profile of this subtype was found to be similar to medullary breast cancer [67,68], indicating a good prognosis and a favorable response to both adjuvant and neoadjuvant therapy [69].

Gene expression profile of the other two subtypes (mesenchymal and mesenchymal stem-like) resemble the chemo-resistant metaplastic breast cancer. The mesenchymal subtype shows elevated levels of genes involved in EMT, cell motility, cellular proliferation and differentiation (*Wnt*, *ALK*, *TGF-β*). On the other hand, the mesenchymal stem-like subtype expresses genes involved in angiogenesis, growth factor pathways along with those regulating cellular proliferation and differentiation (*EGFR*, *PDGFR*, *ERK1/2*, *VEGFR2*) [57]. Moreover, the mesenchymal stem-like subtype shows low-levels of *claudins-3,4,7*; a characteristic similar to the claudin-low subtype [31]. Furthermore, both subtypes (mesenchymal and mesenchymal stem-like) may respond well to PI3K/mTOR inhibitors as well as abl/src inhibitor (dasatinib) [57].

The last known subtype, luminal androgen receptor (LAR), is found to overlap with the molecular apocrine group (“molecular apocrine breast cancer”/MABC) and is enriched in genes regulating hormone signaling, in particular androgen signaling and synthesis (*AR*, *FOXA1*, *KRT18*, *XBP1*) [70]. This subtype displays shorter relapse-free survival and plausible therapeutic targets include flutamide, enzalutamide, bicalutamide [71]. However, the LAR/MABC may not be equivalent to invasive apocrine carcinoma as defined by cancer morphology and steroid receptor profile [72].

Research showed that the basal-like 2 subtype has worst survival, whereas, LAR has the best survival rates. Although, molecular subtypes of TNBC are associated with differences in survival and can potentially contribute in treatment selection, the association of patient race or ethnicity with subtypes of TNBC and clinical outcome still lie nascent. A recent study showed that more than half (53%) of Hispanic women had a significantly higher proportion of basal-like 2 subtype, whereas Asians had a lower proportion (19%) and a higher proportion of LAR (38%) compared to the average proportion across all groups [73]. On the other hand, Asian women had a better overall survival compared to other ethnic groups [73]. These variations across racial and ethnic groups in the subtypes may explain differences in their outcomes. Determining TNBC subtypes can help in understanding the heterogeneity of TNBCs and can pave the way for developing subtype-specific therapies and better predictors of TNBC prognosis for all races and ethnicities.

The Cancer Genome Atlas Research Network (TCGA) used genomic DNA copy number arrays, exome sequencing, mRNA arrays and miRNA sequencing in 76 TNBC patients and identified several mutated genes, the most common being *TP53* (80%), *PIK3CA* (9%), *MLL3* (5%), *AFF2* (4%), *RB1* (4%), and *PTEN* (1%) [58]. Whole genome sequencing analysis of 65 TNBC cases detected six SMGs, of which *TP53* was the most frequently mutated gene. Moreover, clonal frequency analysis identified somatic mutations in *TP53*, *PIK3CA* and *PTEN* dominant in the majority of TNBCs [74]. Several other studies have also confirmed that *TP53* gene as the most commonly mutated gene (65–80%) in TNBC [58,74]; these mutations result in genetic instability and cytogenetic alterations [75]. Research showed that a loss of *TP53* resulted in enhanced metastasis and worse overall survival [76]. Furthermore, the presence of mutations in *TP53* can be a predictor of chemo-resistance in breast cancer [77,78] including neoadjuvant chemotherapy; however, larger prospective studies are needed to further analyze its role as a potential therapeutic target in breast cancer as well as other cancers [79]. The other most common gene involved in breast cancer including TNBC is *BRCA1/2*; more than half of the hereditary TNBC cases (80%) carry mutation in *BRCA1*, while germ-line mutation in *BRCA1* occurs in 15% of TNBC cases [80,81]. Patients lacking *BRCA1/2* function are sensitive to platinum derivatives as well as PARP inhibitors [56]. Several investigations have identified and validated potential biomarkers of genomic instability as a response to platinum-based therapy in TNBC [82].

Recently, a tissue microarray study on African-American women displayed a significant link between TNBC and loss of *PTEN* gene, a negative regulator of the PI3K pathway [83]. They also showed that a loss of *PTEN* activates the mTOR pathway resulting in a high cellular proliferation leading to a more aggressive cancer phenotype and progression [83]. The study implied mTOR inhibitors as potential therapeutic agents. Similar results were found using tissue microarray in Middle Eastern population, where loss of *PTEN* occurred at high frequency in TNBC and was associated with poor prognosis [84]; thus it can be used as a predictive factor for a poor clinical response of neoadjuvant chemotherapy in TNBC [85].

Moreover, African-American women with breast cancer showed increased expression of *p53*, *BRCA1*, *Aurora A*, *Aurora B* and polo-like kinase signaling networks in comparison with European women [38,86]. Additionally, incidence of germline *BRCA1* mutations is relatively low in comparison with women of European descent [38]. Furthermore, compared with African Americans, non-Hispanic, non-Jewish [87,88] and the Ashkenazi-Jewish women [87] had higher rates of deleterious *BRCA1* mutations. Similarly, less than 20% of African-American women had germline mutations in comparison with Caucasian non-Ashkenazi-Jewish women with TNBC who had at least 50% rate of germline *BRCA1* mutations [89], thus, indicating other underlying mechanisms for the onset of TNBC in African-American women. Genes involved in the WNT–β-catenin pathway were significantly deregulated in women of African origin compared with women of European descent, suggesting stimulation of the WNT–β-catenin pathway in the development of the more aggressive phenotype of TNBC in women of African origin [38,90].

Furthermore, phosphatase *INPP4B*, a negative regulator of the PI3K pathway, was found to be lost in TNBC. Loss of *INPP4B* was linked with advanced tumor grade, larger tumor size, a loss of hormone receptors and aggressive tumors. Alterations in *PIK3CA* enhance the PI3K pathway and are present in around 10% of TNBC cases [91]. This data indicates frequent alterations in the PI3K/AKT/mTOR pathway in TNBC and are considered as potential therapeutic targets. *INPP4B* is a distinctive marker for human basal-like carcinoma and can be a potential candidate for treatment using PI3K pathway inhibitors [92]. Nevertheless, initial clinical data from phase I trials using inhibitors did not show any substantial response rates when used as a single agent therapy [93]. A phase 2 clinical trial demonstrated that ipatasertib, an AKT inhibitor, improved the outcomes in a subset of patients with metastatic TNBC when combined with paclitaxel [94]. In addition, development of novel compounds with distinctive specificity and potency targeting different PI3K/AKT/mTOR components and related molecules are under process as they can provide a huge range of toxic profiles and immediate efficiency [94]. Research is now focusing on analyzing possible inhibitors of PI3K/AKT/mTOR for treating TNBC alone or in combination with other drugs [95]. Moreover, drugs targeting other components of the pathway are being developed and include PDK1 inhibitors, SHIP agonists, and heat shock protein inhibitors [93].

Another study identified six differentially expressed genes (*IL32*, *PTX3*, *GATA3*, *TMEM158*, *ETS1* and *MYBL1*) in TNBC, which differentiated a subset of TNBC-25 (25 TNBC samples) from other TNBCs, as well as TNBC from normal-like, luminal A, luminal B and HER2 patient samples [96]. In TNBC patients in Mexico, a gene signature with 9 over-expressed genes (*PRKX/PRKY*, *UGT8*, *HMGA1*, *LPIN1*, *HAPLN3*, *FAM171A1*, *BCL141A*, *FOXC1* and *ANKRD11*) and 1 down-regulated gene (*ANX9*) involved in metabolism was discovered using microarray gene expression profiling, however, further research needs to be conducted in different populations and geographical areas [97]. In parallel, gene expression analysis along with the Gene Set Enrichment Analysis (GSEA) was used to identify the Yin (upregulated pathway in cancer) and Yang (down-regulated pathway in cancer) in TNBC samples. The analysis showed that while, FOXM1 was upregulated, PPARα was downregulated in TNBC; the Yin and Yang pathways allowed categorization of TNBC further into six sub-groups (C1–C6) each having different clinical outcomes, thus providing insight into TNBC heterogeneity; however, further validation for prognosis and treatment is required [98]. Blocking of FOXM1 induces apoptosis and reduces invasiveness and VEGF expression of TNBC cells; impeding FOXM1 along with cisplatin treatment shows synergistic effect. FOXM1 can serve as a potential target for anticancer activity as well as overcoming cisplatin resistance in TNBC [99,100]. Another transcription factor, FOXA1 can play a role in cellular differentiation; thus, overexpression of FOXA1 is associated with a favorable prognosis [101].

Gene expression analysis along with pathway enrichment analysis identified pathways and genes (*SOX8*, *AR*, *C9orf152*, *NRK* and *RAB30*) involved in the onset of TNBC that could be developed as potential therapeutic targets [102]. Two-step genetic screening in TNBC showed loss of *ADNP*, *AP2B1*, *TOMM70A* and *ZNF326* in nude mice, of which further research on *ZNF326*, showed that it regulated tumor cell growth through effects on RNA splicing, epithelial-mesenchymal transition, and cancer stem-cell self-renewal. This study identified novel tumor suppressors in TNBC that can be used as potential targets for therapeutic approach [103]. Loss of expression of these genes lead to cellular migration and invasion (Table 2) and is associated with patient survival [103].

In a Japanese study conducted by Komatsu et al., DNA microarray identified 104 genes that were significantly over-expressed in TNBC and included cancer specific kinases (*NEK2*, *PBK* and *MELK*) as well as genes involved in mitosis (*ASPM* and *CENPK*), which can be developed as molecular targets [104]. Deregulation of *ASPM*, *CENPK*, *MELK*, *NEK2*, *PBK* genes play a role in tumorigenesis and cell cycle regulation; since they induce programmed cell death, therefore, they can be targeted as novel treatment in TNBCs [104]. On the other hand, androgen receptor (AR) regulates cellular proliferation and differentiation; its presence can indicate a good prognosis [105]. Treatment of both LAR and non-LAR TNBC subtypes using AR inhibitors enzalutamide and bicalutamide in in-vitro and xenograft models showed elevated apoptotic rate and loss of proliferation, anchorage-independent growth, migration, and invasion [106,107]. While, the TBCRC011 study, using bicalutamide in AR-positive patients showed a relatively weak response, with a 6-month clinical benefit rate of 19% [108], a MDV3100-11 study using enzalutamide showed higher clinical activity, with a 6-month clinical benefit rate of 28% [109]. Further research aims on explicating the underlying mechanisms of AR therapy resistance and how to classify patients based on the outcome. Further investigations involve use of CYP17 inhibitors or a combination of AR inhibitors with CDK4/CDK6 inhibitors, PI3K inhibitors or neoadjuvant chemotherapy [110]. AR is an easily detectable marker and can aid in classifying TNBC patients who will derive the least clinical benefit from standard chemotherapy. AR-dependent TNBC patients could gain from targeted therapy based on AR antagonists alone or in combination with other chemotherapeutic agents [111].

Furthermore, in China, potential biomarkers (*HORMAD1*, *ELF5*, *KLK6*, *GABRP*, *AGR2*, *AGR3*, *ANKRD30A*, *NME*5 and *CYP4Z3P*) were identified using gene microarray to characterize TNBC [112]. *Anterior Gradient (AGR)-2* and *-3* are involved in cellular migration, transformation, metastasis and apoptosis. While overexpression of *AGR2* indicates bad prognosis, overexpression of *AGR3* can be used as a serum-based biomarker for detecting cancer at early stages [113]. In another study in China, microarray analysis revealed differential gene expression profiles between breast cancer subtypes among which *COL4A2*, *BMF*, *DUSP1*, *FOXA1* and *MLPH* were identified as potential candidate gene targets in TNBCs [114]. Another major study using transcriptome microarrays established a combined mRNA-long non-coding (lnc) RNA signature based on the mRNA species for *FCGR1A*, *RSAD2*, *CHRDL1* and the lncRNA species for *HIF1A-AS2* and *AK124454*. They further demonstrated that *HIF1A-AS2* and *AK124454* enhanced cellular growth and invasion in TNBC cells and contributed to a paclitaxel resistance [115]. Another gene expression analysis study was performed to identify prognostic markers for TNBC; the study found that overexpression of *EOMES*, *RASGRP1* and *SOD2* were associated with better overall survival, while, loss of *FA2H* and *GSPT1* were linked with better overall survival in TNBC [116].

Furthermore, based on a microarray study, other little-known genes in TNBC were identified; two upregulated (*PROM1* and *KLK6*) and seven downregulated (*KRT18*, *GPR160*, *CMBL*, *AGR3*, *CREB3L4*, *CRIP1* and *SDR16C5*) genes that could serve as plausible biomarkers [112]. Moreover, *KRT18* is used to determine poor response to chemotherapy [112].

Bioinformatics analysis in TNBC showed the presence of genes (*AURKA*, *BIRC5*, *BUB1B*, *BUB1*, *CCNB1*, *CDK1*, *KIF11*, *MAD2L1*, *NDC80* and *PLK1*) involved in cellular proliferation; *CCNB1* displayed overexpression and was significantly associated with poor prognosis in TNBC [117]. Although these studies were carried out in South Asian population, different genes were found to be involved in the pathogenesis of TNBC and these could be used as promising therapeutic targets.

Table 1 summarizes list of genes identified in TNBC by gene expression profiling in different geographic regions and Table 2 gives a brief overview of the biological functions of some identified genes in BC.

On the other hand, the initial commercial gene expression signature of BC is MammaPrint^®^ (Agendia, Amsterdam, The Netherlands), measures mRNA of 70 gene expressions as an assay with prognostic value in breast cancer patients. It has been validated for patients with stages I/II and negative or either one or three positive lymph nodes. This gene signature stratifies patients into low-and high-risk groups and identifies patients who can avoid adjuvant chemotherapy [123,124]. Although the stratification is beneficial for ER+ breast cancers, it lacks advantage for ER− cancers, thus making it limited to a substantial proportion of patients [125]. MammaPrint^®^ has been approved by the Food and Drug Administration (FDA) and has been recommended by several guidelines such as St. Gallen′s International Oncology Guidelines for the treatment of early stage breast cancer.

The Oncotype DX^®^ test (Genomic Health, Redwood City, CA, USA) measures 21 gene-expressions (15 tested genes associated with breast cancer plus 6 reference genes). Oncotype DX^®^ test analyzes genes associated with the ER status, proliferating genes, Her2-related genes as well as genes related to cancer invasion. This test provides information whether chemotherapy treatment will be beneficial [126], measures the recurrence risk and classifies them into low-risk, intermediate risk or high risk groups (the Recurrence score is given as a number between 0 and 100) [126]. The Oncotype DX^®^ test may also be utilized for ductal carcinoma in situ (DCIS), the most common form of non-invasive breast carcinoma. This test did not require the FDA approval but has been recommended by various authority bodies and guidelines [127].

The Prediction Analysis of Microarray (PAM) algorithm to a 50-gene set (Prosigna^®^, Stanford, CA, USA) is a 50-gene signature, with an algorithm for the intrinsic molecular classification of breast cancer. It was introduced to improve immunohistochemical and microarray classification. The PAM50 groups breast cancer patients into luminal A, luminal B, HER2 and basal-like [128]. Based on PAM50 score, a phase II trial in metastatic TNBC treated with platinum monotherapy showed an increased trend toward objective response rate in basal versus non-basal TNBC, however results were not statistically significant [129]. Another study had a neoadjuvant setting and involved pretreatment of tumor samples. The results showed and advantage in the addition of carboplatin in all PAM50 subtypes, including non-basal TNBCs [130]. These studies indicated the limited use of available PAM50 assay in managing several TNBC cases. This test is also validated to predict the risk of metastasis for the postmenopausal patients with ER+, HER2-negative, early breast cancer with negative lymph nodes.

The EndoPredict Test (provided by Myriad Genetics, Inc., Salt Lake City, UT, USA), is another genomic test utilized for patients with newly diagnosed, early-stage (node negative), ER-positive and HER2-negative breast cancer. It includes 12 genes: Eight cancer related genes, three RNA reference genes and one DNA reference gene [131]. EndoPredict calculates a risk score called Endopredict score, which can be used with well-established clinicopathologic variables in predicting patients’ outcome. Although the EndoPredict Test has not been routinely approved by the FDA, some authorities such as ASCO suggested its use to assist in the decision-making regarding adjuvant chemotherapy treatment in patients with early-stage, ER–positive, and HER2-negative breast cancer [131].

Breast Cancer Index (provided by BCI, Biotheranostics, Inc., San Diego, CA, USA) is based on the expression of five proliferation-related genes (molecular grade index (MGI)). It gives the 2-gene ratio HOXB13:IL17BR (H:I) in a linear model. The BCI was developed for the decision-making of adjuvant hormonal therapy in postmenopausal women with early stage, ER-positive BC [132].

As indicated, the TNBC subtype is highly heterogeneous and its classification is routinely based on immunohistochemical biomarkers and limited gene signatures (e.g., PAM50 and Lehmann’s system) [29,57]. Although, these are vital prognostic tools, they are frequently applicable to the luminal subtypes and their use as prognostic tools for TNBC has not been validated yet [133]. Hence, there is an urgent need to develop signatures to aid in the early diagnosis and better treatment stratification of the TNBC patients. Today, with the advancement of genomic techniques and assays, developing novel diagnostic and prognostic biomarkers provide further insights into possible therapeutic targets.

In conclusion, it is evident that gene profiling of BC including TNBC in a specific population of different genetic background can play an important role in developing new biomarkers and gene targets for the management of different types of BC and especially TNBC (Figure 1). In addition, it is important to note that a recent AJCC TNM also incorporated the genomic assays discussed above into the current TNM staging system of BC (eighth edition published in 2017) [131]. However, none of the clinically validated gene expression assays has been approved or recommended for TNBC and HER2-negative patients but for ER+ breast cancers. Therefore, further efforts should be made to accomplish this extremely important task and clinically validate gene expression assays for a more proper management of the patients with these aggressive cancers.

In parallel, it is important to highlight that microRNA profiling can also be essential in the development and management of BC and especially TBNC (Figure 1) which is the topic of the following section.

## 4. MicroRNAs (miRNAs) in TNBC

MicroRNAs (miRNAs) belong to the class of small non-coding RNA, measuring around 25nt in length. miRNAs have distinct functions at the post-transcriptional level [134,135]. Since miRNAs are stable in whole blood, plasma, and serum, circulating miRNAs are being studied in healthy controls and BC patients as a potential diagnostic, predictive and prognostic biomarker for the development of therapeutic strategies [136].

miR-30 expression is associated with ER and PR expression while miR-213 and miR-203 expression are linked with tumor stage. In BC, loss of 29 miRNAs was identified when compared with normal breast tissues [137]. Experimental studies have demonstrated the role of miRNAs in the metastatic process, where few miRNAs are either significantly upregulated or downregulated [138].

A recent study on four ethnic groups identified differential expression of 9 miRNAs. In Nigerian patients, significantly higher levels of miR-140-5p, miR-194 and miR-423-5p were seen in BC compared with other ethnic groups [139]. On the other hand, in Indian patients, miR-101 was overexpressed in BCs [139]. Furthermore, in-silico analysis of miR-423-5p showed that AC genotype was associated with Europeans; while, Asians and Africans displayed the CC and AA genotype, respectively [139]. Another study identified 33 previously undescribed miRNA variants, and 31 miRNA containing variants to be differentially expressed between African and non-African populations [140]. Furthermore, a 26-miRNA panel differentiated TNBC between African American and non-Hispanic White women; however, further validation is needed [141]. A study on Lebanese BC patients showed 21 dysregulated miRNAs and 4 miRNAs with different expression patterns in comparison with American patients; plausible cause for these variations could be age of diagnosis or ethnic variation affecting miRNA epigenetic regulation or sequence of miRNA precursors [142]. Nevertheless, variation in miRNA expression in BCs from different ethnic groups can indicate that specific genetic variants in miRNAs may affect breast cancer risk in these groups.

Various miRNAs were linked with EMT and the development of stem-cell properties. These miRNAs included upregulated expression of miR-10b, miR-21, miR-29, miR-9, miR-221/222, miR-373 as well as downregulated expression of miR-145, miR-199a-5p, miR-200 family, miR-203, miR-205 in TNBC [143,144]. In this regard, tristetraprolin, a target for miR-29a, regulates EMT and metastasis in BC [145].

The miR-200 family including miR-200b, suppress cancer cell growth as well as EMT by targeting ZEB1/2, SIP1, BMI1 proteins and inhibiting PKCα [146,147,148,149,150]. The miR-200 expression was lost in TNBC cells in comparison with other subtypes of breast cancer resulting in increased cellular migration and invasion [43,147,148]. In addition, a loss of miR-200 family was observed in mesenchymal-like TNBC human breast cancer cell lines including MDA-MB-231 [151,152]. The loss of miR-206 in TNBC was shown to promote angiogenesis and invasion in both cell-lines as well as tissue samples [153]. Recently, a study in breast cancer cell lines revealed miR-199/miR-214 as a cluster of miRNAs enhancing cellular motility and aggressiveness via proliferation and EMT [154]. A loss of miR-214 increases the aggressiveness of TNBC via proliferation and EMT, as well as promotes cell growth by enhancing the PTEN-PI3K/AKT signaling pathway. Alterations of miR-10b, miR-21, miR-29, miR-145, miR-200 family, miR-203, miR-221/222 were found to be of prognostic value in TNBC patients [143]. A research study by Kim et al. (2011) analyzed the therapeutic effect of miR-145 against breast cancer and found that adenoviral construct of miR-145 (Ad-miR-145) had the potential to inhibit cell growth and motility both in vitro and in vivo [155]. Furthermore, a combined treatment of Ad-miR-145 and 5-FU showed a remarkable anti-tumor activity when compared to treatment by 5-FU alone [155].

Microarray analysis also revealed deregulation (loss) of miR-205 in cells that undergo EMT in TNBC in response to TGF-β [151,156]. MicroRNA expression profiling in TNBC samples revealed low miR-205 indicating its tumor-suppressive role [157]. P53-stimulation leads to loss of miR-205 in TNBC and its re-expression significantly inhibits cell proliferation, cell cycle progression and tumor growth in vivo [156]. Research showed E2F1 and LAMC1, known regulators of cell cycle progression, adhesion, proliferation and migration as experimentally validated targets for miR-205 [156].

Circulating miR-21 distinguished patients with loco-regional disease from those with metastases [158]. miR-21 promotes metastasis of breast cancer cells by targeting *PTEN*, *TIMP1*, *TIMP3*, *PDCD4* [158] which in turn affects the PI3K/AKT/mTOR pathway [159]. In addition, miR-21 sera levels are linked with TNBC phenotype and familial breast cancer along with lymph node metastasis and a higher Ki-67 expression [160,161].

Using qPCR, miR-190a, miR-136-5p, miR-126-5p, miR-135b-5p and miR-182-5p were linked with the pathogenesis of TNBC. MiR-190a plays a tumor-suppressor role preventing metastasis, growth and cell invasion by suppressing VEGF-mediated tumor angiogenesis [162]. On the other hand, miR-135b family plays an oncogenic role regulating the cell cycle, and promoting TNBC cells invasiveness and migration by targeting TGF-beta, WNT and ERBB pathways [163]. A few common genes under the regulation of miR-135b include APC, KLF4, *MAFB*, *CASR*, *PPP2R5C*, *SMAD5*, *LZTS1*, *MID1*, *MTCH2*, *ACVR1B*, *BMPR2*, *TGFBR1*, *IBSP*, *BGLAP*, *RUNX2* and, *SP7* [162]. MiR-34a/c is a tumor suppressor and induces apoptosis in TNBC cells [164,165]; loss of miR-34a/c [164] and miR-940 [166] in TNBC was linked with tumor progression and poor prognosis.

A panel of several miRNAs were also significantly altered in TNBC, indicating their role as useful prognostic and therapeutic factors in TNBC [167,168,169,170]. While miR-135b, miR-105/93-3p, miR-21, miR-17-5p, miR-27a, miR-95-3p were attributed to the onset, progression and metastases of TNBC [163,171,172,173,174,175], another array of miRNAs unraveled to be linked with chemo-resistance [170,176,177,178]. Thus, up-regulation of miR-155-5p, miR-21-3p, miR-181a-5p, miR-181b-5p, miR-183-5p, miR-105/93-3p and loss of miR-181a, miR-10b-5p, miR-451a, miR-125b-5p, miR-31-5p, miR-195-5p and miR-200c were found to be highly associated with promoting chemo-resistance [146,174,176,179,180,181,182]. MiR-27a plays a role in the onset and progression of tumor cells in TNBC and can predict response to radiotherapy and serve as a prognostic marker [175]. Presently, investigations aim to identify miRNA clusters associated with chemoresistance and to help pave the way for the development of more efficient therapies.

MiRNA profiling by next-generation sequencing (NGS) in TNBCs revealed different expression patterns of miRNAs, of which three miRNAs (miR-224-5p, miR-375 and miR-205-5p) can be used to categorize cancers based on their proliferation, invasion and metastasis. Six miRNAs (high let-7d-3p, miR-203b-5p and miR-324-5p; low miR-30a-3p, miR-30a-5p and miR-199a-5p) were significantly related to decreased overall survival while 5 additional miRNAs (high let-7d-3p; low miR-30a-3p, miR-30a-5p, miR-30c-5p and miR-128-3p) were associated with decreased relapse-free survival [173]. Another study demonstrated that loss of miR-30a in TNBC, which suppresses cell invasion and metastasis of the tumor by directly targeting *ROR1*; miR-30a is linked with higher histological grade and lymph node metastasis [183]. Moreover, sequencing identified that loss of miR-4319 in TNBC and presence of miR-4319 was shown to reduce malignant potential of TNBC cells as it suppresses the self-renewal and formation of tumor spheres in TNBC through E2F2 as well as inhibits tumor initiation and metastasis [184]. Deep sequencing along with hierarchical clustering analysis exhibited 25 miRNAs signature to distinguish TNBC from normal breast tissue [185]. Genome-wide miRNA profiling showed a panel of 26 miRNAs to help distinguish TNBC in African-American women from the Non-Hispanic White patients [141].

Lack of miR-603 resulted in high *eEF2K* expression followed by the onset and progression of TNBC [186]. Another miRNA, miR-199a-5p, was found to have a tumor suppressive role in TNBC. High levels of miR-199a-5p in vivo reduced cell motility and invasiveness as well as repressed tumor cell growth [187]. Tissue microarray analysis showed that loss of miR-493 in TNBC patients can be linked with poor disease-free survival, depicting its role as a prognostic factor in TNBC [188]. Using miRNA array analysis, miR-211-5p showed to block proliferation, invasion, migration and metastasis by targeting *SETBP1*; indicating a tumor suppressive role of miR-211-5p in TNBC; [189]. While, miR-148a [190] and miR-629-3p [191] were identified as promoters of lung metastases; while, miR-141 was identified as an enhancer of brain metastasis; suggesting their roles as biomarkers and latent targets of metastases [192].

Studies have also shown presence of upregulated miRNAs in TNBC. The miR-10 family (miR-10a and miR-10b) is involved in both the progression and metastasis of breast cancer [193]. MiR-10b is one such group of miRNAs, highly elevated in TNBC cell lines MDA-MB-231 and SUM1315 compared with normal mammary epithelial cells HMECS and MCF10A [194,195]. miR-10b is significantly upregulated in metastatic breast cancer cells and initiates cell migration and invasion in murine xenograft model of breast cancer by targeting the *HOXD10* gene along with E-cadherin and Tiam1 [196,197,198]. MiR-10b controls cell migration and invasion and regulates the expression of miR-9. MiR-9 is upregulated in TNBC in comparison with the luminal and HER2-enriched breast cancer subtypes [199] and stimulates cell motility and invasion ability by targeting E-cadherin, activating the β-catenin pathway and enhancing VEGF levels [195]. In TNBC, miR-9 was linked with *MYC* amplification, higher tumor grade, as well as significant metastatic potential leading to poor outcome [195,200]. Moreover, elevated miR-105/93-3p enhances the Wnt/βcatenin signaling by downregulation of *SFPR1* leading to chemo-resistance and metastasis [174]. MiR-221/222 [201], miR-761 [202] and miR-373 [165,203,204] are frequently upregulated in TNBC. Research on metastatic samples showed an inverse correlation between miR-373 and CD44; targeting of CD44 by miR-373/520 increases the migratory and invasive ability, both in vitro and in vivo. Clinical metastasis samples also showed an inverse correlation between miR-373 and CD44 expression [204]. High levels of miR-221/222 enhance drug resistance and promote EMT, invasion and cancer cell migration. Additionally, miR-221/222 were also associated with advanced stage, tumor grade and negative hormone receptor status [201,205]. Among Indian women with TNBC, a miRNA signature of 6 different miRNAs (miR-21, miR-221, miR-210, miR-195, miR-145 and let-7a) were associated with an advanced stage, higher tumor grade and negative hormone receptors [205].

miR-21 is the principal miRNA linked with migration and invasion of breast cancer cells and hence plays a critical role in tumor progression and metastasis [206,207]. A report by Iorio et al. (2005) showed that along with miR-125b, miR-145 and miR-155, miR-21 is aberrantly expressed in human breast cancer [137]. Tropomyosin 1 (TPM1) has been discovered as a plausible target of miR-21 [208]. While, miR-21 is inversely associated with *PTEN* expression in BC [209], which is directly linked with TGF-β [210]. Overexpression of miR-21 leads to an aggressive disease status along with higher tumor grade, negative hormone receptor status and ductal phenotype [210]. A recent investigation conducted in Saudi Arabia identified miR-195 in the plasma of TNBC patients [211].

In summary, a large group of miRNAs has been reported to be implicated in TNBC initiation, progression and/or metastasis. These miRNAs can be differentiated based on their functional characterization in TNBC as tumor suppressors and oncogenes. They may also play both diagnostic and predictive roles. Therefore, we believe that miRNA represent as an important target in the management of BC including TNBCs, however, it is important to highlight that genetic backgrounds of different populations have to be carefully examined in order to identify specific miRNAs associated with populations of various ethnicities (Figure 1).

Table 3 below summarizes key miRNAs with their expression levels and biological functions in TNBC.

Despite the array of miRNAs that have been suggested as plausible biomarkers, their use in clinical practice still remains nascent. One of the major reasons being the challenge in miRNA expression profiling; miRNAs are tiny molecules in which family members display a high degree of homology, and absolute miRNA concentrations in body fluids are relatively low [214]. There are several technological advances for using miRNAs as therapeutic tools for cancers. miRNA expression profiles are correlated with genetic subtype and isotype [215]. Biology and characteristic features of miRNAs have been studied among different cancers. Standardizing expression of down-regulated miRNAs or overexpressed miRNAs can aid to re-balance the expression of genes associated in oncogenesis and tumor progression; hence, targeting miRNAs may provide an important therapeutic strategy for human cancer [196,216]. On the other hand, blocking overexpressed miRNAs was accomplished using anti-miRNA oligonucleotides (AMOs), which are complementary to miRNAs. While, generation of down-regulated miRNAs were accomplished using expression systems that use viral or liposomal delivery systems for the vectors [217,218].

Various miRNAs are validated in preclinical tests and are now under further clinical investigation. In 2013, The first miRNA replacement therapy with MRX34—a liposome-formulated miR-34 mimic was carried out. This study underwent human clinical trials for patients with advanced or metastatic liver cancer by intravenous injection [219]. Moreover, to treat different solid carcinomas including lung and prostate cancer, let-7 mimic was developed [220,221]. For hepatitis C, an antagonist of miR-122 was used and tested in phase II clinical trials [222]. Moreover, an investigation by Di Martino et al. [223] proved that either transient expression of miR-34a synthetic mimics or lentivirus-based stable enforced expression of miR-34a, triggered growth inhibition and apoptosis in MM cells in vitro and in vivo without systemic toxicity. Blocking of miRNA-21 using antisense oligonucleotides reduced growth of MCF7 cells by topotecan by around 40% [224]. Similarly, in lung cancer cell lines, inhibition by AG1478 reduced cellular growth [193,225,226]. Recently, MRG-106, an LNA anti-miR of miRNA-155 entered clinical phase I evaluation. Inhibition of miRNA-155 in lymphoma cells reduced proliferation in-vitro [214]. However, there are several challenges including suboptimal delivery, low bioavailability or long-term safety. Research is focusing presently on latent methods including nanoparticles, polymers and virus-based approaches [227]. Nevertheless, and given the important role of miRNA profiling in personalized medicine, we believe that more studies are necessary to elucidate miRNA profile variations in relation with ethnicity.

## 5. Conclusions

In BC, gene-expression-based-assays and the classification of patients have a robust clinical impact and help in individualized therapy and personalized cancer management [228]. Therefore, several gene expression-based assays have been clinically validated and utilized for ER+ but not ER- BCs such as TNBC.

Differential gene expression using microarray profiling on a subset of BC including TN from different geographical regions in comparison to a set of normal/benign breast tumors should be performed to further understand the underlying mechanisms of TNBCs.

Numerous challenges hinder treatment of BC, particularly in TN subtype resulting in a high cancer mortality. Genetic markers of women from different ancestries that predispose them to TNBC have not been entirely elucidated. Therefore, biomarkers for TNBC prognosis of specific ethnicities are urgently needed since they can be used as predictive biomarkers as well as tools for targeted therapy in these populations. In short, discovering combined gene and miRNA signatures of TNBC in different populations and ethnicities could help identify new and specific gene targets for this subgroup of cancers and can be regarded as a fertile ground to accomplish a personalized medicine approach, which is the main objective of modern cancer treatment.

## Figures and Tables

**Table 1:** List of Genes involved in Progression of Triple-Negative Breast Cancer Identified by Gene Expression Profiling.

Gene	Country	Method	Reference
*PTEN*	USA, Middle East	Tissue microarray	[83,84]
*PIK3CA*	USA	Reverse phase protein array	[91]
*ADNP*, *AP2B1*, *TOMM70A*, *ZNF326*	USA	Two-step genetic screening	[103]
*ANKRD11*, *BCL141A*, *FAM17IAI*, *FOXC1*, *HAPLN3*, *HMGT8*, *HMGA1*, *LPIN1*, *PRKX*, *PRKY*, *UGT8*	Mexico	Micro-array gene expression profile	[96]
*FOXM1*, *PPAR*	Canada, United Kingdom	Gene enrichment analysis (GSEA) Gene expression analysis	[98]
*SOX8*, *AR*, *C9/F152*, *EOMES*, *FA2H*, *GSPT1*, *NPK*, *RAB30*, *RASGRP1*, *SOD2*	China	Gene enrichment analysis (GSEA) Gene expression analysis	[102,116]
*BMF*, *COL4A2*, *DUSP1*, *FOXA1*, *FCGR1A*, *HIF1A-AS2*, *MLPH*	China	Microarray analysis	[114]
*RSAD2*, *AK124454*	China	Transcriptome microarrays	[115]
*AGR2*, *AGR3*, *ANKRD30A*, *CMBL*, *CREB3L4*, *CRIP1*, *CYP4Z3P*, *ELF5*, *GABRP*, *GPR160*, *HORMAD1*, *KLK6*, *KRT18*, *NME5*, *PROM1*, *SDR16C5*	China	Gene microarray	[112]
*CCNB1*	GEO database China	Bio-informatics analysis	[117]
*ASPM*, *CENPK*, *MELK*, *NEK2*, *PBK*	Japan	DNA microarray	[104]

**Table 2:** List of Genes and their role in TNBC.

Biological Functions	Genes	References
Cell Proliferation	*PTEN* *INPP4B* *PIK3CA* *FOXM1* *AR* *AGR3* *DUSP1*	[118] [119] [120] [99] [105] [121] [122]
Tumor Metastases and Progression	*FOXM1* *AGR2*	[99] [113,121]
Cell Cycle Regulation	*CCNB1* *ASPM*, *CENPK*, *MELK*, *NEK2*, *PBK* *FOXM1*	[117] [104] [99]
Apoptosis	*DUSP1* *AGR3*	[122] [121]

**Table 3:** List of miRNAs and their Roles in TNBC.

Biological Functions	miRNAs		References
	Stimulate	Inhibit	
**Cell Proliferation**	miR-155-5p, miR-199, miR-761, miR-27a, miR-224-5p, miR-375, miR-205-5p	miR-940, miR-211-5p, miR-148a	[166,173,189,190]
**Tumor Metastases and Progression**	miR-21, miR-21-3p, miR-135b, miR-205-5p, miR-135b-5p, miR-224-5p, miR-375, miR-629-3p, miR-141, miR-10b, miR-105/miR-93-3p, miR-761, miR-181a, miR-181a-5p, miR-181b-5p, miR-183-5p	miR-190a, miR-30a, miR-4319, miR-200, miR-214, miR-31-5p, miR-211-5p, miR-148a, miR-373	[146,147,148,151,154,158,160,161,162,165,166,171,173,174,176,183,184,189,190,191,192,194,196,199,200,202,203,204,212]
**Cell Cycle Regulation**	miR-135b, miR-135b-5p		[163,213]
**Cell Apoptosis**	miR-31-5p	miR-21, miR-23p, miR-27a	[158,160,161,167,171,175,205,212]
**Resistance to Therapy**	miR-21, miR-21-3p, miR-155-5p, miR-195-5p, miR-210, miR-221/222	miR-10b-5p, miR-125b-5p, miR-35p, miR-451a, miR-200c	[146,158,160,161,167,171,176,179,201,205]
**EMT**	miR-155, miR-199, miR-221/222	miR-200, miR-200b, miR-200c, miR-206, miR-373	[146,147,148,149,150,151,157,165,199,203,204]
